# Distal pulmonary epithelial maturation in preterm infants: does the lung of the preterm infant continue its functional pulmonary development postnatally?

**DOI:** 10.25122/jml-2025-0072

**Published:** 2025-05

**Authors:** Raluca Chirculescu, Paul Cristian Balanescu, Gheorghe Peltecu

**Affiliations:** 1 Carol Davila University of Medicine and Pharmacy, Bucharest, Romania; 2 Department of Pathology, Filantropia Clinical Hospital, Bucharest, Romania; 3 Department of Internal Medicine, Colentina Clinical Hospital, Bucharest, Romania; 4 Department of Obstetrics and Gynecology, Ponderas Academic Hospital, Bucharest, Romania

**Keywords:** functional pulmonary maturation, surfactant deficiency, surfactant, Napsin A, premature birth

## Abstract

Disruption of pulmonary development caused by premature birth before the achievement of functional pulmonary maturation culminates in respiratory distress syndrome, primarily due to surfactant deficiency. Furthermore, the severity of this syndrome intensifies, particularly in the case of extremely premature neonates. This investigation aimed to evaluate the presence of postnatal pulmonary functional maturation in premature neonates. In pursuit of this objective, we conducted immunohistochemical assays for surfactant and Napsin A within the pulmonary tissue of 67 preterm neonates, with gestational ages ranging from 23 to 35 weeks, whose lifespans varied between one day and 149 days. The two immunohistochemical markers were evaluated within the pulmonary distal epithelium, and their expression was interpreted in relation to various pre- and postnatal factors. The examination was performed on tissue microarrays, sectioned at 5 micrometers, and the assessment of the immunohistochemical markers was interpreted from photographs captured under the optical microscope. Our investigation revealed that all neonates, regardless of their gestational age or lifespan, demonstrated the presence of surfactant within the pulmonary tissue. The intensity of Napsin A expression exhibited a positive correlation with gestational age, duration of oxygen therapy/mechanical ventilation, administration of antenatal corticosteroids, and maternal infections during pregnancy. In summary, our research demonstrated that mechanical ventilation, through the dynamic process of alveolar distension, promotes surfactant production within the distal lung epithelium. Antenatal treatment with corticosteroids and maternal antenatal infections enhances pulmonary function, facilitating surfactant production.

## INTRODUCTION

Lung development encompasses a phase of structural maturation, during which the bronchial tree and alveolar spaces are established, followed by a subsequent phase of functional maturation wherein the lung epithelial cells acquire distinctive characteristics and the synthesis of surfactant is initiated [1,2]. Disruption of pulmonary development due to premature birth prior to the attainment of functional pulmonary maturation culminates in respiratory distress syndrome, attributable mainly to surfactant deficiency. Furthermore, the severity of this syndrome intensifies, particularly in the case of extremely premature neonates [3].

Although the structural development of the lungs is of indisputable significance, bearing substantial clinical ramifications for premature infants, it is not the focal point of this study. In the following discussion, we shall exclusively address the functional maturation of the preterm lungs.

Pulmonary epithelial differentiation involves the emergence of type I and type II pneumocytes in the distal pulmonary regions [4] and the development of ciliated cells, neuroendocrine cells, and secretory cells within the proximal lung areas [5-7]. While epithelial differentiation in the distal pulmonary regions begins during the canalicular phase of lung development, epithelial maturation in the proximal regions starts earlier during the pseudoglandular phase. Like branching morphogenesis, this complex process evolves progressively from the proximal to the distal regions [4]. In the following sections of this article, we will focus on and discuss epithelial differentiation in the distal regions of the lung, represented by the alveolar epithelium.

At the end of the canalicular phase of pulmonary development, the alveolar epithelium undergoes a remarkable transformation from a cuboidal configuration to a flattened morphology, thereby facilitating the formation of the alveolar-capillary membrane. This epithelium is predominantly characterized by type I pneumocytes, whose primary function is facilitating the gas-exchange process [8]. Concurrently, type II pneumocytes, essential for the biosynthesis of surfactant, initiate their differentiation process by the end of this developmental phase [9]. Although surfactant production begins around the 26^th^ week of gestation, its maturation is not fully realized until after the 35^th^ week [10]. While surfactant production begins early in intrauterine development, the intracellular accumulation of surfactant within lamellar bodies starts to manifest following the 24^th^ week of gestation, with the effective intra-alveolar release of surfactant occurring after the 28^th^ week of gestation [11].

Although specific cellular markers characteristic of alveolar epithelial cells, such as Napsin A, are detectable within the undifferentiated epithelium of pulmonary pseudoglandular structures [12], the distinct morphological and ultrastructural features of the alveolar epithelium arise later, in conjunction with the progression of gestation [13]. Napsin A is an aspartic proteinase that plays an essential role in the maturation of prosurfactant protein B, which is expressed both within the cytoplasm of type II pneumocytes and in the alveolar macrophages [14].

This investigation aimed to evaluate the presence of surfactant and Napsin A within the pulmonary distal epithelium and to interpret their expression in relation to various pre- and postnatal factors, thereby assessing the postnatal degree of functional pulmonary maturation in premature neonates.

## MATERIAL AND METHODS

### Study subjects

The investigation encompassed the use of clinical data of neonates and their maternal counterparts, in addition to paraffin-embedded lung tissue acquired after conducting the necropsy examination of the deceased infants born prematurely between 2017 and 2024. All this information was gathered from two reference maternity wards in Bucharest, Romania, following the affirmative approval of the ethics committees from both medical institutions, as well as the consent of the hospital directors granting access to this medical data.

The primary criteria utilized in the selection of cases incorporated into the study encompassed prematurity and a minimum survival duration of 24 hours. Furthermore, cases diagnosed with pulmonary hypoplasia, regardless of the cause, were excluded from the study.

### Sample size and data collection

After the selection process, 67 subjects were included in the study. These subjects were selected from the databases of the pathology departments at two maternity hospitals in Bucharest. Necropsy examinations were conducted at the time of death, after which representative fragments of tissue from each organ were selected and processed following established protocols. For this investigation, only millimetric fragments of pulmonary tissue were used.

The study considered pertinent neonatal information, including gender, gestational age at birth, lifespan, birth weight and death weight, Apgar score, the necessity for birth resuscitation, duration of oxygen therapy, particularly when administered through mechanical respiratory, support attributable to the important role of positive pressure in the expansion of alveoli, administration of surfactant, antibiotic therapy, caffeine, and immunoglobulin. Additionally, we incorporated post-necropsy data regarding the presence of pulmonary lobar abnormalities, pulmonary fibrosis, or pulmonary emphysema.

Maternal characteristics were also important, and we extracted relevant information from the medical records regarding maternal age, mode of delivery, fetal presentation, number of gestations, prenatal follow-up, antenatal administration of corticosteroids, maternal infections during pregnancy, gestational hypertension, preeclampsia, pre-existing hypertension, gestational diabetes, intrauterine growth restriction, in vitro fertilization, maternal anemia, and stillbirth.

### Work procedures

After the selection of cases, the paraffin blocks were retrieved from the archives. Using a hollow needle, a millimeter-sized specimen of pulmonary tissue was extracted from each paraffin block. The collected specimens were incorporated into two additional paraffin blocks, creating two tissue microarrays designated for their respective medical centers. The precise location of each lung fragment was documented and encoded, ensuring that it could be easily identified during microscopic examination. Following the acquisition of the paraffin-embedded tissue microarray, five-micrometer sections were prepared—one designated for conventional hematoxylin-eosin staining and two for the immunohistochemical analysis of the markers surfactant and Napsin A. For the assessment of pulmonary type II cells, an immunohistochemical analysis was performed utilizing an automated method to determine the presence of surfactant and Napsin A. Surfactant (Zeta ZM124) and Napsin A (Zeta ZR206) were both diluted to a ratio of 1:100.

After sectioning the histological slides, they were placed in a thermostat for 1 hour and subjected to antigen retrieval utilizing PT-link (Dako) for 30 minutes at 97 degrees Celsius. Upon reaching a water temperature of 65 degrees Celsius, the slides were extracted and allowed to rest in the wash buffer for 10 minutes. After the slides reached ambient temperature, they were immersed in a peroxidase-blocking solution for five minutes, followed by three successive buffer washes. The following procedure entailed the application of the specific antibody, which was incubated for twenty minutes, followed by three sequential wash buffers and an additional treatment with HRP for another twenty minutes. This step was followed by an additional series of three consecutive wash buffers. The substrate was prepared in conjunction with DAB and allowed to incubate for five minutes, then underwent three consecutive wash buffers. Ultimately, the counterstaining procedure utilizing hematoxylin was carried out for one minute. The slides underwent a series of dehydrating immersions in progressively concentrated alcohol, starting with a 96% solution and culminating in absolute alcohol, followed by passage through xylene. After the histological slides were mounted, they were ready for examination under the optical microscope.

### Digital image analysis

From each case included in the study, microphotographs were captured at magnifications of 200x and 400x to highlight the cytoplasmic positivity of the immunohistochemical markers for surfactant and Napsin A. The images were obtained at the Filantropia Clinical Hospital in Bucharest, using the Nikon Eclipse Si trinocular optical microscope equipped with a 12.5-megapixel digital camera. The images were then processed employing MShot Digital Imaging Software version 1.1.6.

### Data analysis and interpretation

Ordinal variables, such as surfactant and Napsin A, were assessed by establishing a scale ranging from 0 to 3, depending on the observed intensity. Continuous variables that displayed a normal distribution were represented as mean values ± standard deviations, while nominal variables were characterized by frequencies and percentages. The significance threshold was set at *P* < 0.05, and the Pearson correlation coefficient was used to assess the statistical relationship and association among the data.

All this data, along with neonatal and maternal clinical information, was entered into an Excel database and subsequently analyzed both descriptively and analytically utilizing Microsoft Office Excel 2021 Pro Plus and IBM SPSS software, version 28. The interpretation of the ordinal variables is described in Table 1.

**Table 1:** Interpretation of ordinal variables

The intensity of the reaction	Interpretation description
0	Complete absence.
1	Low-intensity cytoplasmic granular reaction discerned at HPF.
2	Moderate-intensity cytoplasmic granular reaction discerned at HPF.
3	High-intensity cytoplasmic granular reaction discerned at LPF.

HPF, high power field; LPF, low power field.

## RESULTS

The scrutinized cohort consisted exclusively of preterm neonates with gestational ages ranging from 23 to 35 weeks, a minority of whom were female infants (44.8%). Of the cohort examined, 83.6% required neonatal resuscitation interventions at birth, 52.23% demonstrated an Apgar score of less than three at one minute, and 26.86% continued to exhibit a score below three after five minutes. Furthermore, 11.97% of the entire cohort attained an Apgar score exceeding 7 at 1 minute, while approximately one-third (29.85%) recorded a score exceeding seven after 5 minutes. The principal neonatal characteristics of the examined population are outlined in Table 2.

**Table 2:** Characteristics of the neonatal cohort

	Min	Max	Mean	SD
Gestational age at birth*	23	35	28.09	± 3.137
Lifespanª	1	149	21.60	± 33.45
Weight at birth^b^	250	3850	1116.34	± 637.18
Weight at death^b^	250	3670	1409.91	± 843.88
Apgar score 1'	0	9	3.55	± 2.11
Apgar score 5'	1	8	5.07	± 1.71
Oxygen therapyª	1	131	17.90	± 26.52

* : weeks; ª: days; ^b^: grams; ': minutes; Min: Minimum; Max: Maximum; SD: standard deviation.

After analyzing the data presented in Table 2, it becomes apparent that the minimum and maximum birth weights reveal unusual results, necessitating an individual evaluation. The premature neonate, weighing 250 grams, was the second fetus from a dichorionic pregnancy at 25 weeks of gestation. The mother experienced gestational hypertension and thrombophilia, with the severe intrauterine growth restriction attributed to the maternal gestational hypertension. The premature neonate, weighing 3650 grams, was a singleton gestational fetus delivered at 34 weeks of gestation and diagnosed with non-immune fetal hydrops, which elucidates the unexpectedly elevated weight for a preterm infant.

A significant parameter for this study that requires additional clarification is oxygen therapy. It is imperative to highlight that every preterm infant included in this analysis (100%) received one or more forms of oxygen therapy, with 97% (*n* = 65) undergoing mechanical ventilation either intermittent positive-pressure ventilation (IPPV), synchronized intermittent mandatory ventilation (SIMV) or high-frequency oscillatory ventilation (HFOV), while only 3% were administered solely a non-invasive form of oxygen therapy.

Apart from oxygen therapy, the administration of surfactant treatment holds significant relevance in the context of this study. Following the evaluation of this treatment within our study cohort, we determined that 67.16% (*n* = 45) of the neonates included in the investigation received surfactant therapy. Among the remaining 22 subjects who did not undergo surfactant treatment, only seven (31.81%) were preterm infants with a gestational age exceeding 32 weeks.

Upon examining the mothers of the preterm infants included in the study, we observed that 17.91% belonged to extreme age groups, with three of them being under the age of 18 and nine exceeding 40 years. Most pregnancies were single-fetal occurrences, whereas 22.38% (*n* = 15) were multiple pregnancies, two of which were achieved through in vitro fertilization.

More than half of the mothers (53.7%) were primigravida, whereas 22.4% had experienced three or more pregnancies. Notably, two women in this multiparous subgroup had a prior history of stillbirth. Because maternal clinical characteristics can significantly influence fetal well-being, this study, and several parallel analyses of the same cohort, focused on identifying the key maternal factors that may affect fetal lung development and maturation.

One of these factors is maternal infections, and if we scrutinize the prevalence of maternal infections within our study cohort, we observed that 37.31% of the mothers of these preterm newborns reported a history of infections during gestation, with 60% of them being in the second trimester of pregnancy at the time of delivery. Furthermore, 36% of the mothers who reported maternal infections during pregnancy experienced premature rupture of membranes, with a mean duration of 183 hours prior to delivery.

Another significant factor that may exert a beneficial influence on fetal pulmonary maturation by elevating fetal serum cortisol levels is preeclampsia. Of the entire cohort scrutinized, 15 mothers of these premature neonates developed gestational hypertension, with 40% of them subsequently progressing to preeclampsia. Furthermore, 66.66% of premature neonates born to mothers afflicted by preeclampsia exhibited severe intrauterine growth restriction.

The last, yet undeniably essential factor influencing fetal lung maturation is the administration of antenatal corticosteroid therapy. This administration is undertaken at the recommendation of the obstetrician for a pregnant woman having a gestational period of between 24 weeks and 33 weeks and 6 days, who is anticipated to deliver within the forthcoming 7 days. Even though the cohort analyzed consisted of just five premature neonates with a gestational age at birth surpassing 34 completed weeks, fewer than half of the mothers of these infants (43.28%) received antenatal treatment with corticosteroids to promote fetal lung maturation. Furthermore, a concerning 26.86% of these mothers failed to comply with a prenatal follow-up regimen throughout the entirety of their pregnancies. Another plausible explanation for the small proportion of pregnant women who do not receive antenatal corticosteroid treatment may be attributed to emergency deliveries or, perhaps, the sudden onset of labor that precipitates their arrival at the hospital during the expulsion process.

**Figure 1:**
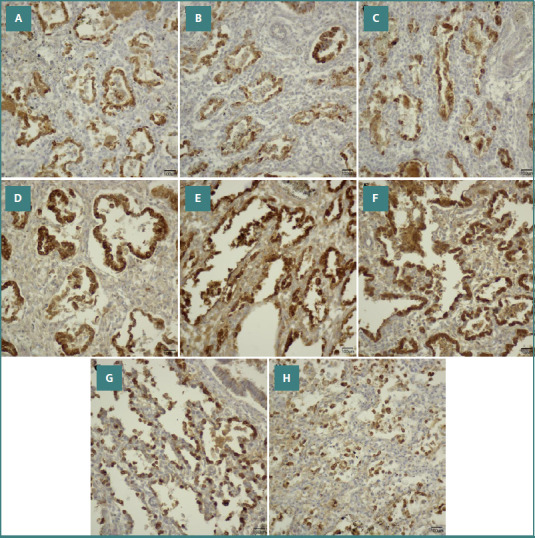
Microscopic images captured at a magnification of 400x, illustrating the immunolabeling for surfactant. A, lung tissue from a 24-week gestation neonate whose mother received antenatal corticosteroid therapy. The membranes ruptured 72 hours before delivery, and the infant required mechanical ventilation for three days, during which exogenous surfactant was administered; survival was three days. B, tissue from a 25-week gestation twin neonate born after an amniotic rupture occurring less than 1 hour before birth. The mother did not receive corticosteroids, and the infant was ventilated for 1.5 days without surfactant therapy; survival was 1.5 days. C, a 25-week gestation infant with intrauterine growth restriction and maternal hypertension. The mother received corticosteroids; mechanical ventilation lasted 3 days, surfactant was given; survival was 3 days. D, tissue from a 26-week gestation infant born to a mother with preeclampsia who did not receive antenatal steroids. The neonate required prolonged ventilation (45 days) and received surfactant therapy; survival was 45 days. E, a 26-week gestation infant whose mother experienced infection during pregnancy and received corticosteroids. Mechanical ventilation continued for 54 days, surfactant was administered, and survival was 54 days. F, lung from a 26-week gestation infant of an infected mother who did not receive steroids; ventilation lasted 93 days, surfactant was given, and survival was 93 days. G, tissue from a 27-week gestation infant delivered after 696 hours of membrane rupture in a mother treated with corticosteroids. The neonate underwent mechanical ventilation for 39 days without surfactant therapy; survival was 39 days. H, a 29-week gestation infant of a mother with gestational hypertension who received antenatal steroids. The infant required 9 days of mechanical ventilation without surfactant administration; survival was 9 days.

In order to evaluate distal pulmonary epithelial differentiation, we examined the presence of surfactant within type II pneumocytes, employing an immunohistochemical technique. After this evaluation, we observed that all cases encompassed in the study (100%), regardless of gestational age at birth, manifested granular cytoplasmic expression of surfactant, with intensities rated at levels 2 or 3 within the alveolar epithelium (Figure 1 A-H).

Because the intensity of the surfactant was moderate to high and consistently present in the pulmonary tissue of all test subjects, this study did not discern a correlation between surfactant intensity and gestational age or lifespan (*P* = 0.78). Furthermore, this investigation did not identify a significant relationship between surfactant intensity in preterm neonates who received surfactant and those who did not undergo this treatment (*P* = 0.89).

**Figure 2:**
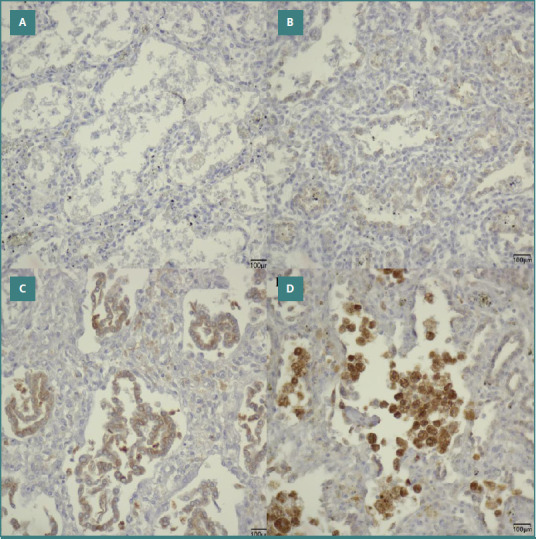
Microscopic images illustrating the immunolabeling for Napsin A (400× magnification). A, Lung tissue was obtained from a 23-week-old neonate who survived for 16 days (score 0). B, Lung tissue was obtained from a 25-week-old neonate who survived for 1.5 days (score 1). C, Lung tissue was obtained from a 29-week-old neonate who survived for 76 days (score 2). D, Lung tissue was obtained from a 27-week-old neonate who survived for 6 days (score 3).

After confirming the presence of surfactant within the pulmonary alveolar epithelium, we evaluated the expression of Napsin A in both the intra-alveolar macrophages and the pulmonary alveolar epithelium. The findings of the study revealed a significant positive correlation between the intensity of Napsin A in intra-alveolar macrophages and gestational age (*P* < 0.001; r = 0.58), as well as between epithelial Napsin A intensity and gestational age (*P* = 0.001; r = 0.31). This suggests that as gestational age advances, the intensity of Napsin A correspondingly escalates (Figure 2 A-D).

In light of the study's objective to determine whether the lung tissue of premature neonates continues its functional maturation after birth, we sought to investigate whether the intensity of Napsin A expression is influenced by lifespan. Specifically, we aimed to evaluate whether premature infants with extended survival exhibited significantly elevated expression of Napsin A. This examination does not support such an assertion; we did not identify any statistically significant relationship between the intensity of epithelial Napsin A and lifespan (*P* = 0.076; r = - 0.18), nor between the intensity of intra-alveolar macrophages and lifespan (*P* = 0.18; r = 0.13).

When we evaluated the intensity of Napsin A epithelial expression alongside the intensity of Napsin A within intra-alveolar macrophages in a cohort of neonates whose mothers received antenatal corticosteroids, and subsequently juxtaposed these findings with those of premature infants born to mothers who did not undergo such treatment, we discerned a statistically significant mean difference between the two groups regarding Napsin A within intra-alveolar macrophages. Thus, preterm neonates born to mothers who did not receive antenatal corticosteroid therapy had a mean reduction of -0.466 in the intensity of Napsin A within intra-alveolar macrophages when compared to preterm neonates whose mothers underwent antenatal corticosteroid treatment (95% CI, -0.91 to -0.14; *P* = 0.022; Table 3).

**Table 3:** Napsin A expression intensity in intra-alveolar macrophages of preterm neonates stratified by antenatal corticosteroid exposure

	No administration of antenatal corticosteroid therapy	Antenatal corticosteroid therapy
Subject number	*n* = 38	*n* = 29
Mean	1.95*	2.41*
SD	± 0.985	± 0.825

*n*, The subject number in absolute value; *: mean intensity expression of Napsin A derived from intra-alveolar macrophages; SD, standard deviation.

It is essential to highlight that this finding was discerned exclusively at the level of Napsin A expression in intra-alveolar macrophages and not within the intraepithelial alveolar Napsin A expression, where no statistical significance was detected (*P* = 0.79).

Another noteworthy observation was made among premature infants conceived by mothers with a documented history of antenatal infections during their gestational period. Among these subjects, there was a distinguished differential of 0.28 units in the intensity of intraepithelial alveolar Napsin A expression when juxtaposed with premature neonates born to mothers without a documented history of infections during pregnancy (95% CI, 0.01-0.54; *P* = 0.038; Table 4).

**Table 4:** Comparison of intraepithelial alveolar Napsin A expression intensity in preterm neonates born to mothers with versus without antenatal infections

	History of infection during pregnancy	No history of infection during pregnancy
No. of subjects	*n* = 25	*n* = 42
Mean	1.16*	0.88*
SD	± 0.473	± 0.550

*n*, The subject number in absolute value; *: Mean of the intensity expression of Napsin A derived from alveolar epithelium; SD, standard deviation.

This correlation was observed exclusively in relation to the intensity of Napsin A expression at the level of the alveolar epithelium, with no analogous correlation detected in the evaluation of Napsin A expression within the intra-alveolar macrophages of subjects whose mothers had a history of infection during pregnancy (*P* = 0.093).

Given the historical link between cervical containment devices (pessaries or cerclage) and increased maternal infection risk, we also examined their impact on fetal lung maturation. This evaluation revealed that premature neonates born to mothers who underwent cervical cerclage or utilized pessary devices had no statistically significant differences within this cohort compared to neonates delivered by mothers who did not necessitate any cervical containment intervention (*P* = 0.93 versus p = 0.41).

Since all these premature neonates underwent a form of oxygen therapy, with 97% being mechanically ventilated during their hospitalization, we aimed to investigate whether mechanical ventilation exerted any influence on the intensity of Napsin A expression in the pulmonary tissue. Following the statistical analysis, we discerned a positive correlation between the duration of mechanical ventilation and the intensity of Napsin A expression in the alveolar epithelium (*P* = 0.002; r = 0.30). This indicated that as the duration of mechanical ventilation increased, there was a concomitant augmentation in the intensity of Napsin expression within the alveolar epithelium. Given that we have earlier clarified that the intensity of Napsin A expression did not increase in direct relation with the prolonged lifespan of these preterm infants, it is highly plausible that the association between mechanical ventilation and the intensity of Napsin A expression is genuine and not merely a confounding variable.

## DISCUSSION

Surfactant, a sophisticated lipoprotein complex, plays an indispensable role in the mechanics of respiration by reducing the surface tension of the alveoli and preventing pulmonary collapse during exhalation [15,16]. The composition of surfactant is predominantly lipidic, characterized by a ratio of 9:1 between lipids and proteins, with phospholipids serving as the principal component; among these, phosphatidylcholine is the most significant [15]. The protein composition constitutes 10% of the surfactant structure and encompasses four distinct surfactant proteins, designated by the letters A through D. Surfactant proteins A and D are essential components of the innate immune response in the pulmonary system, while surfactant proteins B and C fulfill the indispensable role of reducing surface tension in the alveoli [17,18]. The focal or diffuse positivity for surfactant observed throughout the cohort examined in our study, irrespective of gestational age at birth, aligns with existing literature indicating that the synthesis of surfactant within type II pneumocytes commences prior to 24 weeks of gestation. Furthermore, the intra-alveolar surfactant, or more precisely, certain components of its lipid constituents, undergo a recycling process within the type II pneumocytes. In other words, through the mechanism of endocytosis, type II pneumocytes assimilate some of the surfactant components, which are subsequently reconstituted intracellularly in the form of lamellar bodies. The residual components of the surfactant that are not incorporated intracellularly will either be recycled extracellularly or subjected to degradation by alveolar macrophages [15,19]. Given that a significant proportion of these neonates receive exogenous surfactant to enhance respiratory functionality, it is highly probable that this substance, or at least a portion of it, will subsequently enter the intra- or extracellular recycling pathways of the surfactant. Although the presence of the surfactant was detected in the distal regions of the pulmonary tissue of these preterm infants, the immunohistochemical examination conducted in this study does not provide information about the quality of the lipoprotein structure of the surfactant. It is well established that the surfactant synthesized in the lung tissue of preterm neonates exhibits a distinct composition in both lipid and protein content. The entire cycle of surfactant—from its biosynthesis to its recycling—occurs at a markedly slower rate compared to that of full-term newborns [19]. For all these reasons, we have determined that a mere assessment of surfactant expression within the alveolar epithelium is inadequate for elucidating the extent of epithelial differentiation as well as the postnatal functionality of lung tissue in these preterm neonates.

Napsin A is a member of the aspartic proteinase family, expressed in type II pneumocytes and alveolar macrophages, and it plays an important role in processing prosurfactant protein B within human pulmonary tissue [20,21]. Surfactant protein B is synthesized in Clara cells and type II pneumocytes in a preproprotein form and subsequently undergoes maturation through enzymatic cleavage. This conversion is exclusively facilitated in type II pneumocytes, where Napsin A plays a critical role in this process [22,23]. Given that Napsin A can be detected intracellularly prior to the emergence of surfactant tissue expression, commencing from week 16 of gestation, we shall employ this immunohistochemical marker as an indicator for identifying type II pneumocytes and will interpret its activity in accordance with the intensity of its expression.

An interesting aspect that we discerned was the statistically significant increase in the expression intensity of Napsin A, both within the alveolar epithelium and in the intra-alveolar macrophages, concurrent with advancing gestational age. This observation implies that as the distal alveolar epithelium matures, the intensity of Napsin A expression correspondingly increases. Therefore, this increased expression of Napsin A may indicate that the enzymatic activity of this protease is higher with increasing gestational age, or it may suggest that the substrate (surfactant) upon which this enzyme exerts its action has become more abundant, or potentially both. Although we would have anticipated achieving a similar outcome, our assessment of Napsin A intensity expression in relation to the lifespan of these preterm newborns, some of whom survived for over 30 days, revealed no statistically significant correlation between these two parameters. This observation raises the intriguing question of whether the intracellular enzymatic systems in these preterm infants maintain a low activity rate consistent with their gestational age at birth, or whether the substrate (surfactant) upon which this enzyme operates remains at insufficient levels, or perhaps both.

Because oxygen therapy is among the most extensively utilized interventions within the preterm infant population, we sought to ascertain whether the intensity of Napsin A expression in type II pneumocytes is influenced in any significant manner by this treatment. The observed positive correlation, particularly the increase in Napsin A expression intensity concomitant with the prolonged duration of oxygen therapy/mechanical ventilation, can be interpreted, albeit indirectly, through the mechanical expansion of pulmonary airspaces initiated by the introduction of oxygen into the lungs rather than through a direct influence of oxygen on the alveolar epithelium. Over time, research has demonstrated that alveolar mechanical distention triggers the release of intraalveolar surfactant by enhancing the merger of lamellar body membranes with the cellular membranes of type II pneumocytes, thereby promoting the discharge of their contents into the alveolar space [19,24]. Stimulation of surfactant secretion through the distension of type II pneumocytes in cellular cultures has been elucidated since the 1990s by Wirtz. Furthermore, Arold *et al*. disclosed in their analysis that the transient stretching of type II pneumocytes in cell cultures promotes surfactant secretion, whereas prolonged distension of these cells, similar to in vivo conditions, is associated with the inhibition of surfactant secretion [25]. Based on the findings from our research regarding the increase in Napsin A expression in conjunction with the duration of oxygen therapy/mechanical ventilation, we speculate that modern oxygen therapy techniques have improved their effectiveness, and that the volutrauma resulting from such interventions now carries far less clinical significance than it did in previous years. Furthermore, it is important to underline that volutrauma occurs due to mechanical ventilation and is not present in non-invasive forms of oxygen therapy.

Although we would have anticipated observing a statistically significant correlation between the intensity of Napsin A expression and the lifespan of these premature neonates, our study did not yield such findings. Nevertheless, it is noteworthy that the *P* value approached statistical significance (*P* = 0.076), which may suggest that the sample size included in the study was insufficient, thereby diminishing its statistical power.

Glucocorticoids play an essential role in the maturation of fetal lungs by facilitating the differentiation of alveolar epithelial cells and enhancing the production of surfactant [16,26]. During intrauterine development, fetal glucocorticoid levels commence a gradual ascent starting at 35 weeks of gestation, at which point the surfactant system achieves its complete development [10,27]. Prior to 35 weeks of gestation, the fetal organism synthesizes a limited quantity of corticosteroids, while maternal steroids are unable to cross the placental barrier due to their inactivation by 11beta-hydroxysteroid dehydrogenase [16]. Therefore, the antenatal administration of corticosteroids to mothers at high risk for preterm birth becomes critically significant and constitutes an essential element of standard care practices [28,29].

Antenatal maternal infections are associated with an increased risk of preterm birth; nevertheless, they are also linked to improved lung development and a decreased incidence of respiratory distress syndrome. This phenomenon prompted the hypothesis that inflammation induces functional pulmonary maturation, accompanied by increased surfactant synthesis. Consequently, numerous animal models of chorioamnionitis have been developed over time to thoroughly examine the validity of this hypothesis [30]. The data currently available in the literature exhibit a lack of uniformity; some analyses indeed indicate an association with a reduction in the occurrence of respiratory distress syndrome. A potential mechanism by which antenatal inflammation facilitates lung maturation may involve pro-inflammatory cytokines that enhance the production of surfactant proteins [31]. In our investigation, the intensity of Napsin A expression was markedly elevated in both the cohort of preterm neonates born to mothers who received antenatal corticotherapy and in the group of premature infants born to mothers with a history of maternal infection. What is notably distinct, however, is that the intensity of Napsin A expression in the intraalveolar macrophages was statistically significantly higher among premature infants born to mothers who received antenatal corticosteroid treatment, without achieving statistical significance regarding Napsin A expression in the alveolar epithelium. Conversely, the intensity of Napsin A expression in the alveolar epithelium was statistically significantly elevated among newborns whose mothers had a history of infections during pregnancy, with no corresponding statistical significance observed in the intraalveolar macrophages. However, the distinction between Napsin A expression in the alveolar epithelium and that in the intra-alveolar macrophages may not hold significant relevance, as intra-alveolar macrophages possess Napsin A due to its phagocytosis resulting from the degradation of the alveolar epithelium. What is essential to highlight, however, is that both the administration of antenatal corticosteroids and the association with antenatal maternal infections are linked with an improvement in the functional maturation of the pulmonary systems in these preterm neonates.

### Limitations

By far, the most significant limitation of this study was that, in conjunction with the immunohistochemical assessment of surfactant, the relative proportions of the lipoprotein constituents of the surfactant were not evaluated to ascertain whether they resembled those observed in preterm infants or if their composition had undergone modifications. A second limitation of the study lies in the fact that the parameters examined were not reported at the postmenstrual age. A third potential limitation of our study lies in the relatively modest sample size, which may adversely affect the statistical power of our findings.

## CONCLUSION

Disruption of pulmonary development caused by premature birth can profoundly impair respiratory function. The identification of surfactant within pulmonary tissue can be discerned at early gestational stages (23–24 weeks of gestation); however, when evaluated in isolation, it failed to yield substantial insight into the degree of functional pulmonary maturation. Napsin A analysis indicated that its intensity of expression amplifies in correlation with gestational age and remains unaffected by lifespan. The intensity of Napsin A expression was positively correlated with the duration of mechanical ventilation, suggesting that mechanical alveolar distension enhances the synthesis of surfactant. Both antenatal corticosteroid therapy and maternal infections during pregnancy enhanced the functional maturation of the lungs in preterm infants; our findings demonstrated a correlation between these parameters and the intensity of Napsin A expression.
